# Epidermal Nevus Following Thermal Burn Injury: A Pediatric Case of Wolf's Isotopic Response

**DOI:** 10.7759/cureus.115956

**Published:** 2026-09-08

**Authors:** Sarah E Rashdan, Erica Teng

**Affiliations:** 1 Dermatology, Baylor College of Medicine, Houston, USA

**Keywords:** burn scar, cutaneous trauma, epidermal nevus, immunocompromised district, pediatric dermatology, thermal burn, verrucous papules, wolf isotopic response

## Abstract

Wolf's isotopic response is defined as the appearance of a new, unrelated dermatosis within the borders of a healed prior skin injury. We report a seven-year-old boy who developed multiple verrucous papules confined to a healed partial-thickness thermal burn on the anterior trunk, with histopathologic findings consistent with epidermal nevus. The clear temporal and spatial relationship between skin injury and lesion development is suggestive of an isotopic response occurring within an immunocompromised cutaneous district, although causation cannot be definitively established. To our knowledge, based on a review of the available English-language literature, this case of epidermal nevus arising within a healed thermal burn expands the spectrum of burn-associated isotopic response.

## Introduction

Wolf's isotopic response is defined as the appearance of a new dermatosis within the locus of a completely healed, prior skin injury [1]. This is distinct from the Koebner phenomenon, wherein trauma triggers lesions of an existing or prior dermatosis. This phenomenon is clinically important because it suggests that skin may remain biologically altered even after complete visible healing. Proposed mechanisms include local immune dysregulation and formation of an immunocompromised district. Although classically described after herpes zoster, burn-induced cases have also been reported [2,3]. Recognizing non-herpetic triggers is important because isotopic responses may otherwise be overlooked when lesions arise in sites attributed to scarring or post-traumatic change. Isotopic responses have subsequently been reported following a variety of cutaneous insults including burns, trauma, radiation exposure, and chronic lymphedema. In burn scars, persistent changes in local immune surveillance, tissue remodeling, and neurovascular signaling may create a permissive environment for later cutaneous disease. This case adds to the limited literature describing benign proliferative dermatoses as potential manifestations of post-burn isotopic response.

## Case presentation

A seven-year-old boy presented with a three-year history of mildly pruritic papules on the trunk. At 18 months of age, he had sustained a partial-thickness thermal burn involving 9% of the total body surface area of the anterior trunk from spilled hot coffee (Figure 1). Acute management by emergency medicine and plastic surgery services included debridement, topical wound care, and pain management, resulting in complete healing devoid of early hypertrophic changes. The overlying skin remained stable until warty papules developed within the margins of the prior injury three years later, at four years of age. The lesions increased in number over the next year before stabilizing. There were no cutaneous lesions at birth, before the trauma, or anywhere else on the body. The prior burn scar was largely flat and soft without hypertrophy beyond the areas occupied by the papules. Review of systems was unremarkable, without findings of epidermal nevus syndrome.

**Figure 1:**
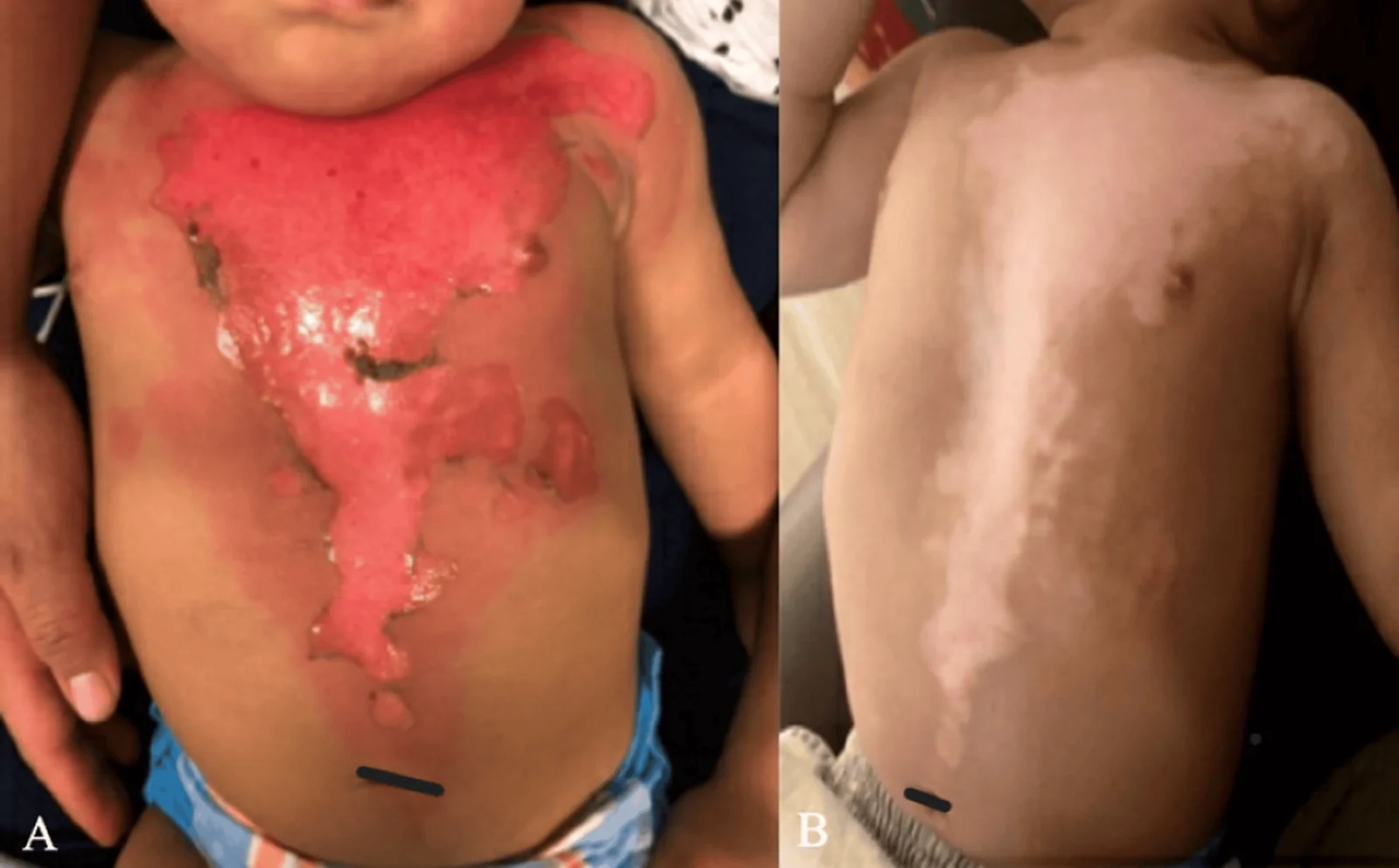
Partial-thickness thermal burn at 18 months of age. (A) Anterior trunk involving 9% of the total body surface area. (B) Complete epithelialization without hypertrophic changes.

On physical examination, there were multiple skin-colored to brown, soft, pedunculated, and verrucous papules on the left anterior chest extending to the abdomen (Figure 2). The distribution of lesions corresponded to the boundaries of the previous injury, with sparing of the surrounding uninjured skin. Portions of the eruption appeared to follow Blaschko lines. A shave biopsy of a lesion in the left clavicular region revealed epithelial fragments with acanthosis and papillomatosis, with superficial perivascular lymphocytic infiltrate consistent with epidermal nevus. Koilocytotic change, significant cytologic atypia, and other features suggestive of viral wart or malignant proliferation were not identified.

**Figure 2:**
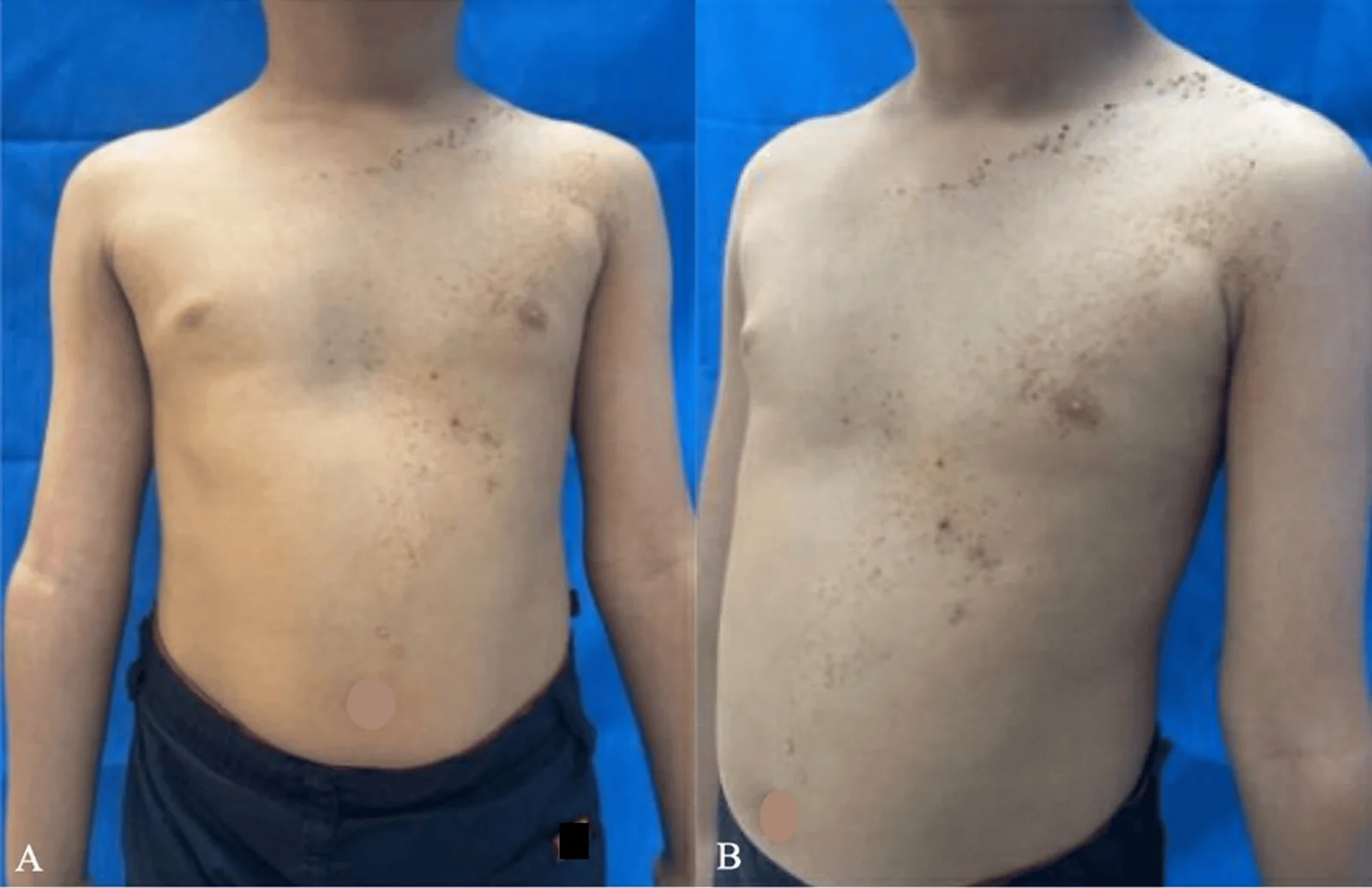
Epidermal nevus within healed burn scar in a seven-year-old boy. (A) Multiple, grouped verrucous and pedunculated papules that arose three years after burn injury. (B) Similar lesions following the boundaries of previous trauma.

## Discussion

Our observation illustrates a clear temporal and spatial relationship between skin injury and subsequent development of epidermal nevi limited to the injured site after complete epithelialization. Histopathology supported a diagnosis of epidermal nevus rather than scar tissue. The absence of additional lesions or associated neurologic, ocular, or skeletal abnormalities supported a localized, non-syndromic epidermal nevus [4]. Verruca vulgaris, fibroepithelial polyps, reactive epidermal hyperplasia, and seborrheic keratosis were considered in the differential diagnosis. However, clinicopathologic correlation favored epidermal nevus based on the lesion distribution, patient age, and histopathologic findings.

The presentation highlights a distinct pathogenic question since epidermal nevi typically arise from mosaic activating mutations in keratinocyte signaling genes and are therefore developmental anomalies rather than acquired lesions [4]. Because epidermal nevi are generally associated with postzygotic mosaic mutations, it is unlikely that the burn-induced genetic changes occurred de novo. The partially Blaschkoid appearance of the eruption raises the possibility that a previously inapparent mosaic lesion was present before injury and became clinically evident following local tissue remodeling. This phenomenon can be explained by the concept of isotopic response via the establishment of an immunocompromised district, a localized field where immune surveillance, vascular architecture, and neuroimmune signaling remain permanently deranged long after clinical healing [5]. A similar mechanism has been implicated in eruptive melanocytic nevi, inflammatory dermatoses, and granulomatous reactions, which suggests that burn scars may create a permissive environment for secondary cutaneous growth [5].

## Conclusions

This observation expands the spectrum of post-burn cutaneous disorders and is suggestive of isotopic response by documenting an epidermal nevus arising within a healed burn scar. Recognizing this presentation is clinically important to prevent misdiagnosis as reactive or neoplastic changes. For clinicians, the key diagnostic clue is not morphology alone, but the delayed emergence of a new lesion type within a sharply localized area of prior injury. This further supports the possibility that previously injured skin can serve as a site of secondary cutaneous disorders. While the temporal and spatial association is suggestive of an isotopic response occurring within an immunocompromised cutaneous district, alternative explanations, including the unmasking of a previously inapparent mosaic lesion, cannot be excluded. In similar cases, clinicopathologic correlation is essential because burn-associated papules may clinically mimic benign, infectious, reactive, or neoplastic entities. Greater awareness of this pattern may help guide appropriate biopsy and avoid unnecessary treatment based on an incorrect presumed diagnosis.
